# Fine needle aspirates comprehensively sample intrahepatic immunity

**DOI:** 10.1136/gutjnl-2018-317071

**Published:** 2018-11-28

**Authors:** Upkar S Gill, Laura J Pallett, Niclas Thomas, Alice R Burton, Amit A Patel, Simon Yona, Patrick T F Kennedy, Mala K Maini

**Affiliations:** 1 Barts Liver Centre, Blizard Institute, Barts and The London School of Medicine and Dentistry, Queen Mary University of London, London, UK; 2 Division of Infection and Immunity, Institute of Immunity and Transplantation, University College London, London, UK; 3 Division of Medicine, University College London, London, UK

**Keywords:** fine needle aspirate, liver biopsy, hepatitis B virus, tissue-resident immunity, hepatocytes, intrahepatic-immune monitoring, HBV-specific T cells

## Abstract

**Objective:**

In order to refine new therapeutic strategies in the pipeline for HBV cure, evaluation of virological and immunological changes compartmentalised at the site of infection will be required. We therefore investigated if liver fine needle aspirates (FNAs) could comprehensively sample the local immune landscape in parallel with viable hepatocytes.

**Design:**

Matched blood, liver biopsy and FNAs from 28 patients with HBV and 15 without viral infection were analysed using 16-colour multiparameter flow cytometry.

**Results:**

The proportion of CD4 T, CD8 T, Mucosal Associated Invariant T cell (MAIT), Natural Killer (NK) and B cells identified by FNA correlated with that in liver biopsies from the same donors. Populations of Programmed Death-1 (PD-1)^hi^CD39^hi^ tissue-resident memory CD8 T cells (CD69^+^CD103^+^) and liver-resident NK cells (CXCR6^+^T-bet^lo^Eomes^hi^), were identified by both FNA and liver biopsy, and not seen in the blood. Crucially, HBV-specific T cells could be identified by FNAs at similar frequencies to biopsies and enriched compared with blood. FNAs could simultaneously identify populations of myeloid cells and live hepatocytes expressing albumin, Scavenger Receptor class B type 1 (SR-B1), Programmed Death-Ligand 1 (PD-L1), whereas hepatocytes were poorly viable after the processing required for liver biopsies.

**Conclusion:**

We demonstrate for the first time that FNAs identify a range of intrahepatic immune cells including locally resident sentinel HBV-specific T cells and NK cells, together with PD-L1-expressing hepatocytes. In addition, we provide a scoring tool to estimate the extent to which an individual FNA has reliably sampled intrahepatic populations rather than contaminating blood. The broad profiling achieved by this less invasive, rapid technique makes it suitable for longitudinal monitoring of the liver to optimise new therapies for HBV.

**Significance of this study**

**What is already known on this subject?**

- Many important immune cell types accumulate preferentially within the intrahepatic compartment.
- There are also liver-resident populations of NK cells and memory T cells, including HBV-specific CD8 providing sentinel front-line immunosurveillance, that cannot be sampled in the blood.
- Liver fine needle aspirates (FNAs) have been shown to sample intrahepatic immunity, including in one case an HBV-specific T cell response, but this modality of liver sampling has not been widely adopted; whether FNAs can be used to investigate tissue-resident T and NK cells, and to what extent they recapitulate the immune profiles obtained by biopsies, is unknown.
- FNAs have also been used for flow cytometric analysis of HBV-infected hepatocytes; parallel analysis of hepatocytes with the immune compartment from the same FNA has not been reported.

**What are the new findings?**

- Sixteen-colour flow cytometric analysis of 43 paired FNAs and biopsies reveals that FNAs capture all immune cell types tested including liver-resident subsets, in a manner that is proportionally representative of biopsies.
- Analysis of a single cell type (eg, CXCR6^+^ NK cells) can be used to estimate how faithfully a given FNA represents the full intrahepatic immune spectrum.
- FNAs consistently allow analysis of intrahepatic HBV-specific CD4 and CD8 T cells, and their functionality, making them ideal for longitudinal monitoring of novel HBV immunotherapies.
- Comprehensive immune profiling can be combined with hepatocyte phenotyping from the same FNA, to optimise analysis of host/pathogen and immune/target cell interactions (including PD-1/PD-L1).

**Significance of this study**

**How might it impact on clinical practice in the foreseeable future?**

- Our findings underscore the value of FNAs as a less invasive alternative to liver biopsies, well suited to longitudinal monitoring of the intrahepatic immune compartment in parallel with hepatocytes.
- We derive an algorithm that can be used to ‘score’ FNAs to allow their standardisation in clinical studies.
- Our data should facilitate the adoption of FNAs in early phase trials for the functional cure of HBV as well as prompting their reconsideration for flow-cytometric monitoring of other liver diseases.

## Introduction

Chronic hepatitis B virus (HBV) is estimated to affect 280 million people worldwide and continues to kill more than 700 000 a year.^1^ Existing antivirals can rarely achieve sustained off-treatment responses but new therapies are in development, aiming to achieve a ‘functional cure’.^2^ This is defined by Hepatitis B Surface Antigen (HBsAg) loss and represents the situation where the majority of infection is cleared and residual intrahepatic HBV covalently closed circular DNA (cccDNA) is kept under tight immune control, as seen in adults achieving natural resolution. A range of direct-acting antivirals and immunomodulatory approaches are being developed and will need careful evaluation as single and combined agents to treat patients in different phases of infection.^3–6^ Thorough virological and immunological studies will be required in early phase trials of new therapies in order to fully understand their mechanism of action in vivo. This will inform the rational selection of biomarkers of responsiveness, the development of drug modifications and selection of combinations. Evaluation of immune boosting will clearly be critical for immunomodulatory therapies but will also be important in the case of antiviral agents, which may indirectly induce some immune reconstitution, for example as a result of a reduction in HBV antigen presentation.^3^

Although many useful immunological insights into HBV pathogenesis have been made by studying peripheral blood, it has become increasingly clear that a large proportion of relevant responses are enriched in the liver. In addition, it has come to light that tissue-resident immune subsets play vital roles in front-line immunosurveillance in the liver and other organs; these cannot be sampled in the blood.^7–9^ We have recently shown that a large proportion of HBV-specific CD8 T cell responses have a tissue-resident phenotype, resulting in their compartmentalisation in the liver.^7^ To achieve functional cure of HBV it will be necessary to boost these locally acting immune responses, which are adapted to survive the hostile liver environment and mount rapid antiviral defence. The liver is also enriched with a number of innate-like populations such as mucosal associated invariant T (MAIT) cells and natural killer (NK) cells; we and others have recently shown that the latter also has a specialised liver-resident component.^10–14^ In addition to these immunological features unique to the liver niche, hepatic sampling also has major advantages over peripheral blood for accurately assessing HBV replication, integration and cccDNA persistence.^5 15–17^

Liver biopsy allows a valuable assessment of intrahepatic immunological and virological, as well as histological, features in HBV infection, but is frequently now replaced with non-invasive monitoring.^5^ Fine needle aspirates (FNAs) could provide a rapid and better-tolerated alternative, such that repeated longitudinal sampling becomes feasible, as recently demonstrated in patients with HCV.^18^ The use of FNA to obtain immune infiltrates or hepatocytes for flow cytometric assessment was pioneered in HBV infection by the Janssen group more than a decade ago.^19^ They showed that HBsAg-expressing, and less frequently hepatitis B core antigen (HBcAg)-expressing, hepatocytes can be detected by flow cytometry of FNAs.^20^ This group also showed that an HBV-specific CD8 T cell response directed against the core-18–27 epitope could be detected in the FNA from a human leucocyte antigen (HLA)-A2^+^ patient.^19^ We therefore postulated that FNAs are well suited to monitor the intrahepatic response to novel therapeutic regimens for HBV infection. To address this, we compared the capacity of FNAs versus biopsies to sample a range of intrahepatic immune cell types, particularly focusing on recently defined liver-resident T cell and NK cell subsets and other liver-enriched populations such as MAIT cells. We showed that HBV-specific T cells directed against a panel of epitopes could be detected equally efficiently by FNA or biopsy. Since a concern with FNAs is their potential contamination with blood, we evaluated the utility of different immune markers to predict how well a sample represents the composition of a biopsy and developed this into a tool for future studies. In addition, we examined whether it was possible to stain immune populations, including myeloid cells, and hepatocytes from the same sample, to allow future integration of information on host immune responses with pathogen-infected target cells.

## Materials and methods

### Patients

Subjects included in this study were undergoing percutaneous liver biopsy for diagnostic purposes; tissue surplus to diagnostic requirements was used in tandem to an FNA and blood obtained at the same visit. Written informed consent was obtained from all patients. Twenty-eight patients with chronic hepatitis B (CHB), spanning the disease phases in line with recent international guidelines^21^ were included; envelope Antigen (eAg)-positive chronic hepatitis; n=3 (of which one patient sampled on antiviral therapy), eAg-negative chronic infection; n=3, eAg negative chronic hepatitis; n=22 (of which three patients sampled on antiviral therapy). In addition 15 patients with non-viral liver disease were included. Additional details and clinical characteristics of patients sampled are included in online supplementary methods and supplementary tables S1A,B.

10.1136/gutjnl-2018-317071.supp1 Supplementary file 1

### Sample collection and cell isolation

For the FNAs, a 22-gauge spinal needle with an internal trocar in situ was inserted along the anaesthetised tract to the edge of the liver capsule. On expiration the needle was inserted a further 2–3 cm into the liver parenchyma. The internal trocar was then removed and a 10 mL syringe filled with cold Roswell Park Memorial Institute (RPMI)-1640 medium (Sigma-Aldrich) attached to the needle. This was then aspirated with gentle negative pressure as the needle was withdrawn 1–2 cm but still while remaining in the liver parenchyma using a ‘fanning technique’ as previously described in endoscopic ultrasound (EUS).^22 23^ The needle was removed and fresh media drawn into the syringe to ensure the entire aspirate passed into the syringe. The syringe was detached and the trocar replaced along with the needle being inserted through the same anaesthetised tissue but orientated in a slightly different direction and the aspiration method repeated as above. The contents of the syringe were then promptly passed into a 50 mL Falcon tube containing RPMI and placed on ice prior to transfer for processing. Any aspirate with frank blood staining was discarded. The liver biopsy was then performed immediately afterwards according to previously published guidelines.^24^

Peripheral Blood Mononuclear cells (PBMCs) were isolated from heparinised blood by density centrifugation using Pancoll (Pan Biotech). Intrahepatic leucocytes (IHLs) from biopsy tissue were isolated by mechanical disruption using cell scrapers without further processing (debris removed by passing single cell suspension through 70 µM cell strainers (Greiner)). The isolation of IHL from FNA has previously been described.^18–20 25^ In brief, samples were centrifuged; the remaining cell pellet was resuspended in 2 mL of red blood cell lysis buffer (Biolegend) for 5 min on ice, prior to staining. All samples were used immediately.

### Flow cytometry and analysis of HBV-specific T cells

Multiparameter flow cytometry was used for phenotypic and functional analysis along with detection of HBV-specific T cells following dextramer staining or peptide stimulation of PBMC and IHL from the FNA and biopsy tissue as previously described.^7^ (For full details of experimental methods see online supplementary methods.)

### Analytical methods: predictive model

For principal component analysis (PCA), the first dimension of the new projection (PC1) contained a linear combination of all the measured subset frequencies from the original data set, with the coefficients chosen to maximise the variance across this dimension. Subsequent Principal Components (PCs) contained progressively less of the variance; the first two PCs were visualised in a two-dimensional plot. To model the probability of a binary response (ie, whether an FNA sample was more ‘liver-like’ or ‘blood-like’) logistic regression was used. Using this approach, the probability of the binary response was assumed to be related to the input variables using the following equation:

Probability (y)=1/(1+e^(β0 +β1 x1 +β2 x2 + …)^, where y is the binary response variable (FNA sample to determine as ‘liver-like’ or ‘blood-like’), x1, x2,… are the values of the independent variables (lymphocytic subset frequencies), and the coefficients β0, β1, β2,… are determined from the training data set.

The probability p(y), p(y) e (0,1), was used to predict the binary outcome, based on the values of the input variables. Finally, each classification model was evaluated in turn using bootstrapping. In this approach, a set of ‘n’ selections was made from a modified data set containing subset frequencies from the biopsy and blood samples only with replacement. The logistic regression model was trained using these samples and tested on the samples that were not selected, with model accuracy determined on these test samples being recorded. This process was repeated for 1000 iterations to provide confidence intervals (CIs) on model accuracy. Once model accuracy was determined, the FNA samples were used as an input for the model of choice (ie, the best predictive model) to evaluate a probability score of how ‘liver-like’ the sampling method was on a patient-by-patient basis. All analyses used for data visualisation and the predictive modelling were performed using Python. The PCA and logistic regression analyses were implemented using scikit-learn, and the graphical visualisations were produced using matplotlib and seaborn packages.

### FNA tool

The equations obtained from the predicative model for frequency of CXCR6^+^ NK cells and/or CD8 T_RM_ (CD69^+^CD103^+^) have been used to formulate an ‘FNA tool’ for users to determine how ‘liver-like’ their FNA sample is. This FNA tool can be accessed via the link: http://www.ucl.ac.uk/maini-group/software. 

### Statistical analysis

Statistical analyses were performed in Prism (GraphPad) using appropriate methods as indicated in each figure legend. Statistical tests used were the following: Wilcoxon signed-rank *t* test, Mann-Whitney U test, Friedman test (analysis of variance; ANOVA) with a Dunn’s post hoc test for pairwise multiple comparisons between each group, or a Spearman’s rank correlation coefficient. Where significant the differences were marked on the appropriate figures. All tests were carried out as two-tailed tests and for all tests significance levels were defined as *p<0.05, **p<0.01, ***p<0.001 and ****p<0.0001.

## Results

### A broad composition of lymphocytes can be obtained by FNA sampling

**Figure 1:**
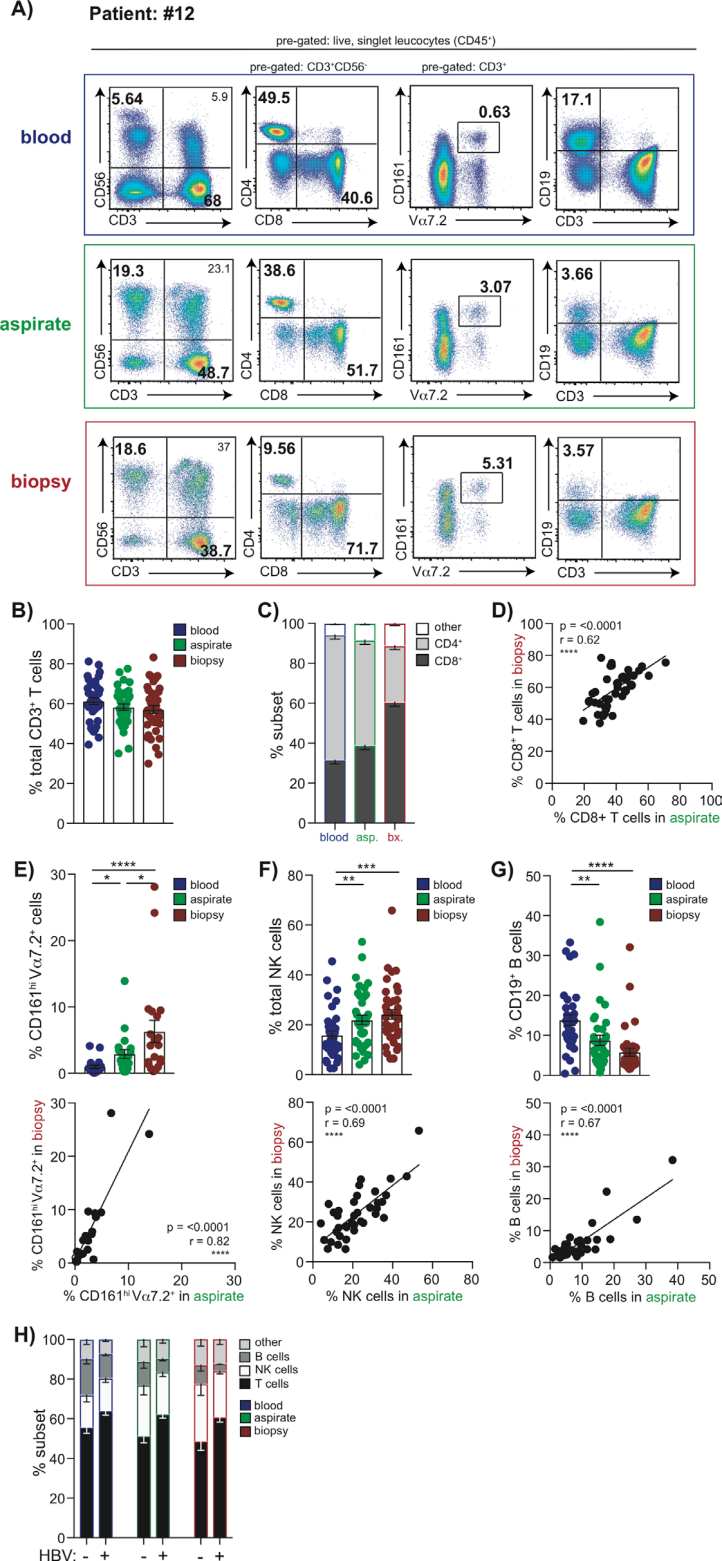
Global lymphocyte profiling of matched blood, FNA and liver biopsy samples. Multiparameter flow cytometric analysis of matched samples from blood (blue), FNA (green) and liver biopsy tissue (red) from the same individuals analysed in parallel. (A) Representative sequential gating strategy used to identify T cell, NK cell, MAIT cell and B cell subpopulations, pregated on live, singlet CD45 expressing leucocytes. Summary data of the frequency of (B) global CD3^+^ T cells, (C) CD4^+^ and CD8^+^ T cells using each sampling method (n=37). (D) Correlation of intrahepatic CD8^+^ T cell frequencies in FNA samples compared with biopsies. Summary frequencies and corresponding correlative analysis of FNA versus biopsy comparing intrahepatic populations of (E) MAIT cells (CD3^+^CD161^hi^V7.2α^+^; n=20), (F) NK cells (CD3^−^CD56^+^; n=37) and (G) B cells (CD3^−^CD19^+^; n=36). (H) Summary lymphocyte profiles of blood with corresponding FNA and biopsy comparing HBV-infected patients (n=22) versus non-HBV infected subjects (n=14). Error bars indicate means±SEM; p values were determined by a Friedman test (ANOVA) with a Dunn’s post hoc test for multiple comparisons, Spearman’s rank correlation coefficient or Mann-Whitney U-test; significant changes marked with asterisks, *p<0.05; **p<0.01; ***p<0.001, ****p<0.0001. ANOVA, analysis of variance; FNA, fine needle aspirate.

Patients having a diagnostic liver biopsy were invited to simultaneously undergo an FNA and venesection. Where surplus tissue was available from liver biopsy, this was compared with FNAs and peripheral blood from the same time point in a total of 43 patients. Using 16-colour flow cytometry we were able to analyse a broad composition of lymphocyte subsets (within the CD45^+^ leucocyte gate) including T (CD4 and CD8), MAIT, NK and B cells (figure 1A). The frequency of total CD3 T cells was not significantly different when comparing blood, FNA and liver biopsy (figure 1B). However, as previously reported CD8 T cells were enriched relative to CD4 T cells within the intrahepatic compartment compared with the peripheral CD3 T cell pool (figure 1C). This CD8 T cell enrichment was less evident in FNAs than in biopsies but their frequencies by these two liver sampling methods were significantly correlated (figure 1D). Innate-like lymphocytes, in particular MAITs, are now known to make up a sizeable proportion of intrahepatic CD3 T cells.^26–28^ In a subset of subjects, we therefore analysed MAIT frequencies by staining for coexpression of their invariant T cell receptor (TCR) Vα7.2 with high CD161 expression (figure 1A,E). MAITs were significantly enriched in the liver compartment and we observed a striking correlation in frequencies detected within an individual by FNA versus biopsy (figure 1E). A similar liver enrichment was seen for NK cells (CD3^−^CD56^+^), which also showed a robust correlation between FNA and biopsy frequencies (figure 1A,F). B cells (CD3^−^CD19^+^) showed the converse pattern, with lower frequencies in the liver than blood, whether sampled by FNA or biopsy, and good congruence between the two methods (figure 1A,G).

We then examined whether any differences were detectable in the immune composition of FNAs taken from patients with HBV infection compared with other liver diseases (online supplementary tables S1A,B). We found that the proportion of T cells was increased (p=0.007), with a concomitant decrease in the percentage of B cells (p=0.018), in HBV compared with non-HBV liver disease (figure 1H). These HBV-specific differences in the hierarchy of different intrahepatic immune cell frequencies were detectable in both FNAs and biopsies, validating the capacity of FNAs to detect novel immunobiological features.

### Liver-resident T cell and NK cell subsets are contained within FNAs

**Figure 2:**
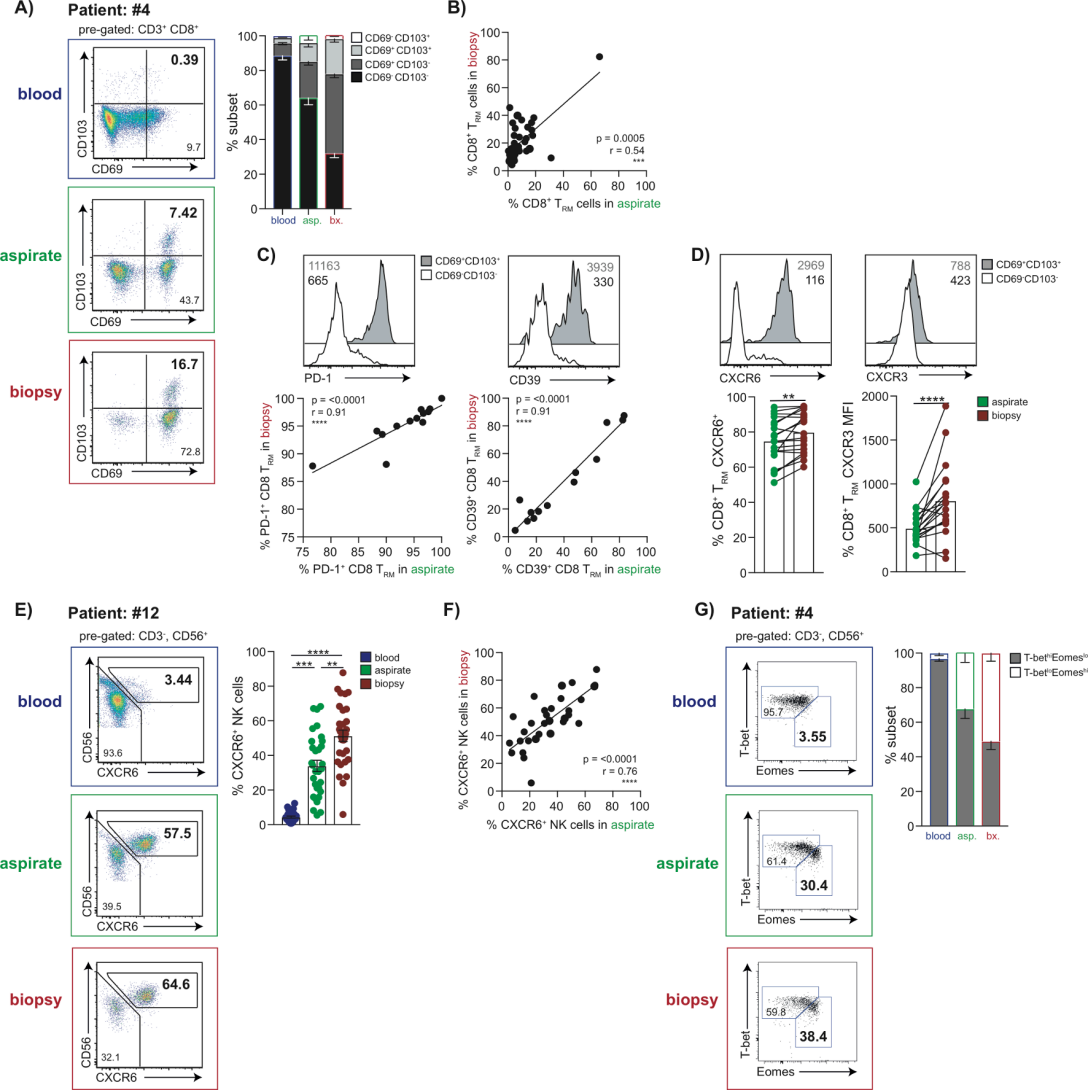
Identification of liver-resident T cell and NK cell populations by FNA compared with liver biopsy. (A) Representative flow cytometry plots depicting non-resident/liver-infiltrating CD8^+^ T cells (CD69^−^CD103^−^), CD69 single positive and liver-resident CD8^+^ T cells (CD69^+^CD103^+^CD8^+^ T_RM_) in matched blood, FNA and liver biopsy tissue and summary data (n=37) of the frequency of each subset. (B) Correlation of CD8^+^T_RM_ frequencies in FNAs and biopsy samples. (C) Representative histograms of PD-1 and CD39 expression on CD8^+^T_RM_ versus non-resident subsets and correlation of %PD-1 and %CD39 on CD8^+^T_RM_ in FNAs and biopsies (n=13). (D) Representative histograms of CXCR6 and CXCR3 expression on CD8^+^ T_RM_ versus non-resident subsets and correlation of their expression on CD8^+^ T_RM_ in FNAs and biopsies (n=20). (E) Representative plots showing CXCR6^−^ and CXCR6^+^ fractions of intrahepatic NK cells and summary data of frequency of %CXCR6^+^ liver-resident NK cells in blood, FNA and liver biopsy (n=32). (F) Correlation of CXCR6^+^ NK cell frequencies in FNAs and biopsies (n=32). (G) Representative plots of T-bet and Eomes expression on CXCR6^+^ NK cells and summary data of the proportion of liver-resident T-bet^lo^Eomes^hi^ versus liver-infiltrating T-bet^hi^Eomes^lo^ NK cells in blood, FNAs and biopsies (n=10). Error bars indicate means±SEM; p values were determined by a Friedman test (ANOVA) with a Dunn’s post hoc test for multiple comparisons, Wilcoxon signed-rank *t* test or Spearman’s rank correlation coefficient; significant changes marked with asterisks, *p<0.05; **p<0.01; ***p<0.001, ****p<0.0001. ANOVA, analysis of variance; FNA, fine needle aspirate.

Next, we investigated whether it was feasible to use FNAs to study specialised tissue-resident T and NK cells that we have recently shown are compartmentalised in the liver.^7 10^ Both FNAs and biopsies contained CD8 T cells with a CD69^+^CD103^+^ tissue-residency phenotype (CD8 T_RM_), that were absent from matched blood samples; although these were at lower frequencies in FNAs (p<0.0001, Wilcoxon test, figure 2A), they were proportional to biopsies (figure 2B). Similarly, the CD69 single positive subset, which contains a large additional fraction of liver-resident T cells and some that can recirculate, was enriched in biopsies more than FNAs (figure 2A). Despite these discrepancies in frequencies, we found that the CD8 T_RM_ sampled in FNAs showed the programmed death-1(PD-1)^hi^CD39^hi^ phenotype characteristic of liver CD8 T_RM_, with proportions correlating tightly with those seen in matched biopsies (figure 2C). These data indicated that FNAs can reliably sample prototypic liver CD8 T_RM_. Nevertheless, the fact that CD8 T_RM_ were obtained at lower frequencies in FNAs raised the possibility that some may have migrated deeper within the liver parenchyma, rendering them only accessible on tissue maceration. We therefore compared their expression of two liver-homing chemokines which we had shown to be hallmarks of liver CD8 T_RM_; those in FNAs had markedly lower levels of CXCR3 and lower levels of CXCR6 than in biopsies (figure 2D).

CXCR6 expression also marks a population of T-bet^lo^Eomes^hi^ liver-resident NK cells that have been shown to be long-lived and unable to recirculate.^10 14^ Frequencies of CXCR6^+^ NK cells were significantly higher, and closely correlated, in FNAs and biopsies compared with blood (figure 2E,F). Using the more definitive T-bet^lo^Eomes^hi^ transcription profile, we confirmed that these were absent from blood and present in similar proportions in FNAs and biopsies (figure 2G).

### Detection of HBV-specific T cells by FNA sampling

**Figure 3:**
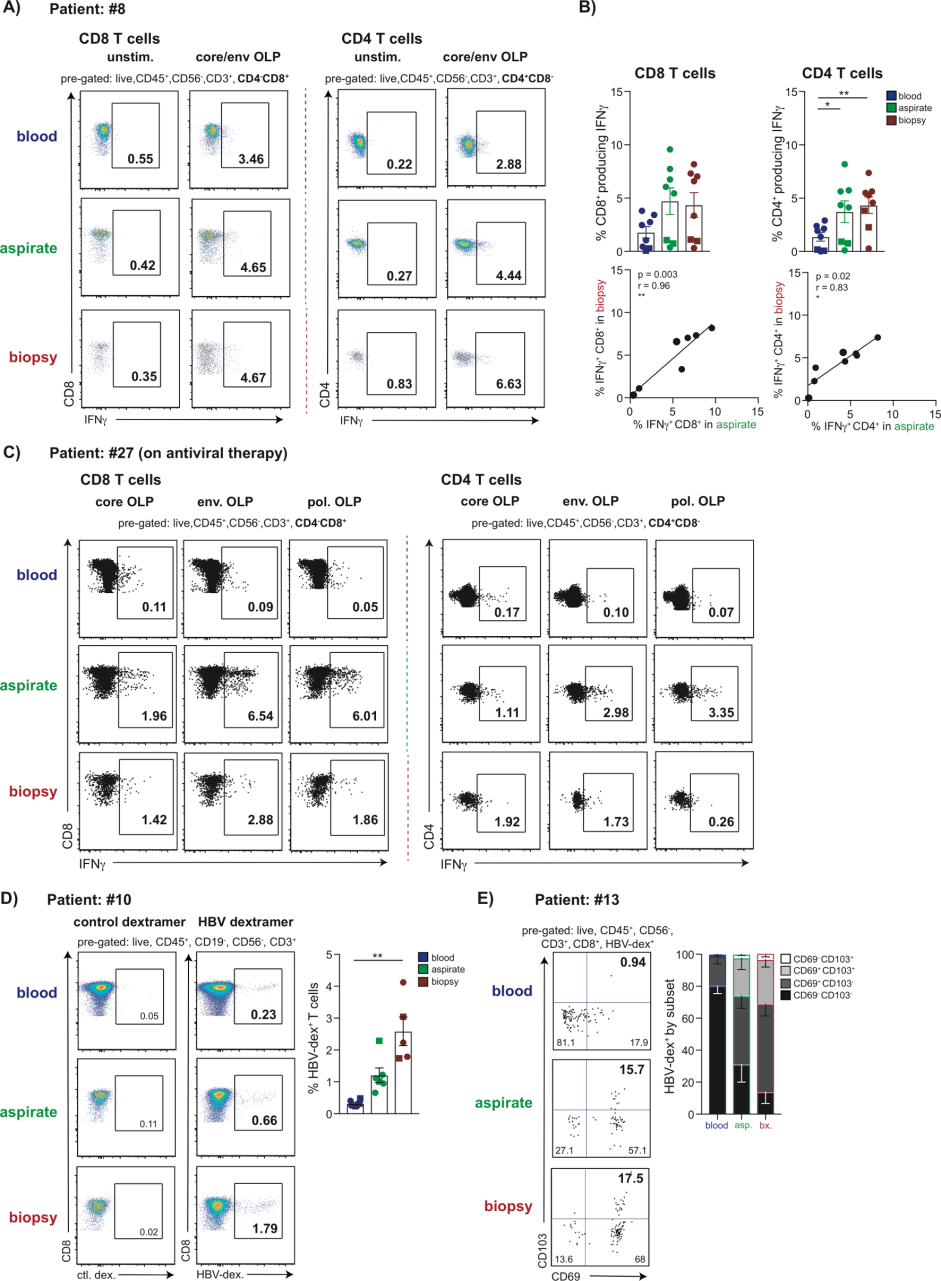
Detection of HBV-specific T cell populations in FNA and liver biopsy samples. (A) Representative plots showing the frequency of HBV-specific CD3^+^CD4^-^CD8^+^ and CD3^+^CD4^+^CD8^-^ T cells (%IFN-γ presented minus paired unstimulated control) matched blood, FNA and liver biopsy tissue, measured by the production of IFN-γ by ICS following 16 hours stimulation with OLP spanning the core and envelope proteins. (B) Summary data of the frequency of HBV-specific CD4 and CD8 T cells (n=8) and their correlation in FNA and biopsy samples. (C) Representative plots showing the frequency of HBV-specific CD3^+^CD4^-^CD8^+^and CD3^+^CD4^+^CD8^-^ T cells in matched blood, FNA and liver biopsy tissue, measured by the production of IFN-γ by ICS following 16 hours stimulation with OLP spanning the core, envelope and polymerase proteins in a virally suppressed patient on antiviral therapy (%IFN-γ presented minus paired unstimulated control). (D) Representative plots and summary data (n=6 for blood and FNA; n=5 for biopsy) of ex vivo staining with a panel of HLA-A2/HBV peptide dextramers (gated using an HLA-A2 restricted control dextramer loaded with an irrelevant peptide). (E) Representative plots and summary data (n=5) depicting the proportion of HBV-specific CD8^+^ T cells identified by ex vivo dextramer staining expressing residency markers CD69 and CD103. Circles in summary data depict treatment-naïve patients and squares indicate patients analysed during antiviral therapy. Error bars indicate means±SEM; p values were determined by a Wilcoxon signed-rank *t* test; significant changes marked with asterisks,*p<0.05; **p<0.01; ***p<0.001, ****p<0.0001. FNA, fine needle aspirate; ICS, intracellular cytokine staining; OLP, overlapping peptide

To analyse functional HBV-specific CD4 and CD8 T cells at the site of infection, we stimulated FNA and biopsy extracts with overlapping peptides (OLPs) spanning core and envelope proteins and tested for interferon (IFN)-γ responses by intracellular cytokine staining (ICS) (figure 3A). IFN-γ-producing HBV-specific CD4 and CD8 T cells were observed at similar, and tightly correlated, frequencies in FNA and liver biopsy specimens, which tended to be enriched compared with blood (figure 3B). We then tested whether FNAs would allow more detailed dissection of specificity, by dividing samples into separate wells containing OLPs from HBV core, envelope and polymerase proteins. There were insufficient events for reliable interpretation of individual specificities from either FNA or biopsy from patients with high viral loads (online supplementary figure S1A). However, a patient with viral load well suppressed on antiviral therapy for >5 years had robust CD4 and CD8 T cell responses detected against separate core, polymerase and envelope peptide pools in both FNA and biopsy samples but not blood, illustrating the utility of liver sampling for future immunomonitoring (figure 3C).

10.1136/gutjnl-2018-317071.supp2 Supplementary file 2

Identification of HBV-specific CD8 T cells with a panel of HLA-A2/HBV-peptide dextramers has the advantage of allowing ex vivo phenotyping. By dividing an FNA and biopsy sample, we investigated whether it was possible to use half for ex vivo HLA-A2-peptide dextramer analysis and the other for detection of functional responses by ICS. Stimulation with peptides covering the same seven immunodominant HLA-A2-restricted epitopes as in the dextramer panel showed similar frequency responses detectable by these two methods in a paired FNA and biopsy (online supplementary figure S1B,C). Further analysis of FNAs and biopsies showed that sufficient HLA-A2-peptide dextramer-stained CD8 T cells could be stained (figure 3D) to allow assessment of their tissue-residency phenotype using CD69 ±CD103, as described above for global CD8 T cells (figure 3D).^7^ Whereas HBV-specific CD8 T cells in the blood had a small percentage expressing CD69 and no CD103^+^CD69^+^, those in FNAs and biopsies were both predominantly single-positive or double-positive CD8 T_RM_, consistent with their compartmentalisation in the infected liver (figure 3E).

### FNA sampling allows simultaneous detection of hepatocytes and leucocytes

**Figure 4:**
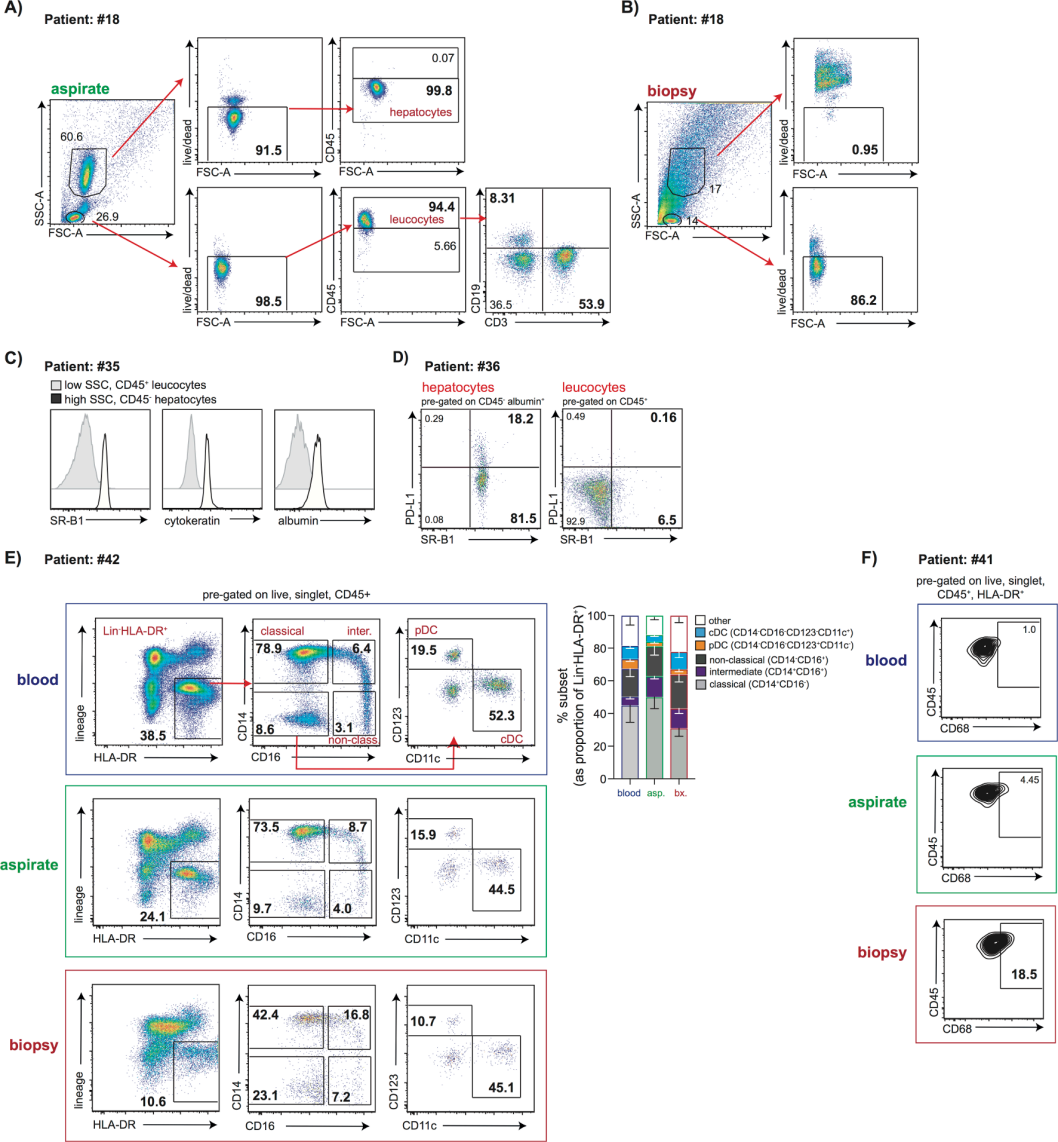
Using FNA samples to analyse viable hepatocytes and leucocytes in parallel. Representative gating strategy used to distinguish viable hepatocytes and leucocytes in the same staining panel using forward and side scatter, a live-dead stain and CD45 in (A) an FNA sample and (B) liver biopsy sample. The example shows additional staining for global B (CD19^+^) and T (CD3^+^) cells. (C) Representative histograms showing SR-B1, cytokeratin and albumin expression, respectively, on hepatocytes (high SSC, CD45^−^; black open histograms) compared with leucocytes (low SSC, CD45^+^; grey filled histograms). (D) Representative flow cytometry plots of PD-L1 expression on CD45^-^ albumin^+^SR-B1^+^hepatocytes compared with CD45^+^ leucocytes by FNA sampling. (E) Representative sequential gating strategy along with summary data (n=5) showing mononuclear phagocyte populations in blood, FNA and liver biopsy samples. Pregated on live, singlet CD45^+^ leucocytes, lineage(CD3,CD19,CD20,CD56,CD66b)^−^ HLA-DR^+^, populations of classical (CD14^+^CD16^−^), intermediate (CD14^+^CD16^+^) and non-classical (CD14^lo^CD16^+^) subsets. Populations of dendritic cells (DCs) identified as CD14^−^CD16^−^ populations; pDC (plasmatocytoid DC) identified as CD123^+^CD11c^−^ and cDC (classical DC) as CD123^−^CD11c^+^. (F) Flow cytometry plot indicating CD68^+^ mononuclear phagocyte (pregated on live, singlet, CD45^+^, HLA-DR^+^) in blood, FNA and liver biopsy. FNA, fine needle aspirate; SSC, side scatter.

We next tested the possibility to simultaneously analyse hepatocytes in conjunction with lymphocyte or myeloid subsets from FNAs. In addition to the classical leucocyte gate set on live CD45^+^ cells, we were able to gate a distinct population of live cells with the increased Side Scatter-Area (SSC-A) and lack of CD45 expression characteristic of hepatocytes (figure 4A). By contrast, the mechanical disruption required to process biopsies meant that only a small proportion of cells falling within the expected hepatocyte gate were viable (figure 4B). To verify the identity of putative hepatocytes in FNA, we showed that they expressed scavenger receptor class B type 1 (SR-B1) as well as cytokeratin and albumin; in contrast, the CD45^+ ^leucocyte gate demonstrated negative expression of these markers (figure 4C). We were able to simultaneously identify viable hepatocytes and leucocytes in all 10 FNAs where this was attempted; additional markers could be included to examine immune cell types of interest within the same panel (eg, B cell and T cell stains shown in figure 4A). Importantly hepatocytes could also be stained for additional markers of relevance to pathogenesis and immunotherapy; in all three cases tested it was possible to visualise a population of programmed death-ligand 1 (PD-L1)-expressing cells restricted to the hepatocyte gate (CD45^-^ albumin^+^SR-B1^+^, figure 4D).

In addition to using a panel that allowed simultaneous staining of hepatocytes and leucocytes, the FNA could be split, with half the sample giving adequate cell yields for hepatocyte staining, leaving the other half for immunological analyses requiring a more extensive antibody staining panel or peptide stimulation for HBV-specific T cells (online supplementary tables S1A,B and S2). Furthermore, myeloid cells including mononuclear phagocyte subsets (classical, intermediate, non-classical)^29 30^ and dendritic cell subsets, broadly divided into plasmacytoid and classical,^31^ could be extracted and identified from FNAs as well as from biopsies (gating strategy and representative example with summary data of five samples in figure 4E). While a firm strategy is yet to be established to identify human liver-resident Kupffer cells, it may be possible to identify these in FNAs, as suggested by the presence of a CD68^+^ mononuclear phagocyte population at a higher frequency in FNA compared with blood, and further enriched in biopsies (figure 4F).

### An algorithm to assess FNA sampling of the intrahepatic compartment

To test the extent to which immune cell types sampled by FNAs contained some representations from blood as well as liver, we compared correlations between blood and FNAs or blood and biopsies (online supplementary figure S2A). Global CD8 T , MAIT , NK  and B cell frequencies in FNAs correlated with those in blood (online supplementary figure S2A), as they had with matched biopsies (figure 1D–G). More surprisingly, peripheral frequencies of MAITs and B cells also correlated convincingly with those in liver biopsies, suggesting their intrahepatic frequencies may be adequately predicted by blood sampling (online supplementary figure S2B). By contrast, the very low frequencies of cells with a ‘liver-resident’ CD8 T cell profile (CD69^+^CD103^+ ^CD8 T_RM_) in blood did not correlate with proportions found in FNAs or biopsies (online supplementary figure S2C). Similarly the low frequency HBV-specific CD8 T cell responses detectable in blood did not correlate with frequencies in FNAs or biopsies (online supplementary figure S2D), in line with our finding that the latter mainly have a tissue-resident phenotype, providing useful confirmation of the value of liver sampling. CXCR6^+^ NK cells are mainly sequestered in the liver but the small proportion sampled in the blood correlated with frequencies in FNAs or biopsies; in contrast T-bet^lo^Eomes^hi^ liver-resident NK cells did not show any correlation between blood and FNAs or biopsies, again suggesting that the blood cannot accurately sample liver-resident populations (online supplementary figure S2E). These findings suggested that blood frequencies can be used to estimate intrahepatic frequencies of some global immune cell types but not of liver-resident and HBV-specific T cells.

10.1136/gutjnl-2018-317071.supp3 Supplementary file 3

A potential concern with liver FNAs is contamination of the sample with subcapsular blood or difficulty aspirating some highly adherent cell types that may be better sampled by tissue maceration. In line with this, FNA sampling underestimated the frequencies of some intrahepatic immune populations quantitated in a liver biopsy within the same individual. To make FNAs more applicable in the clinical setting, we therefore developed a predictive model to enable investigators to assess the reliability of an individual FNA at fully representing the immune profile that would be expected to be obtained from liver tissue had the patient instead had a biopsy.

**Figure 5:**
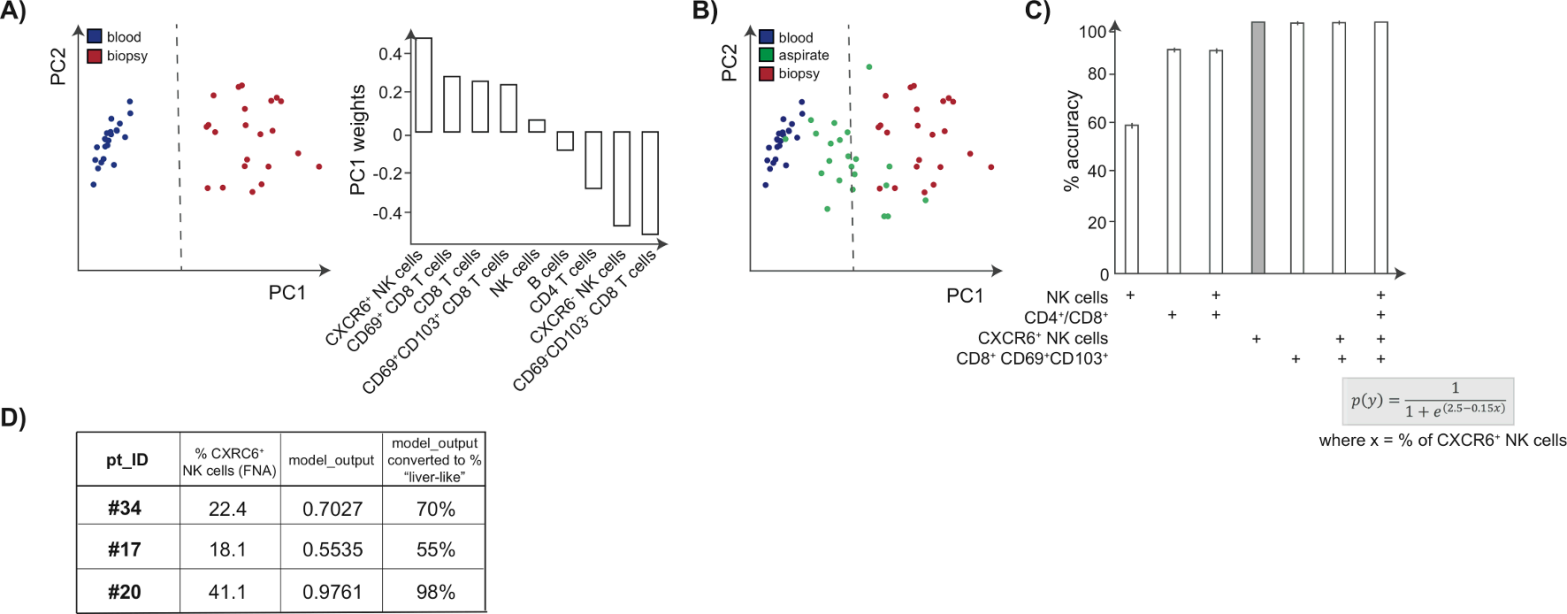
Frequencies of CXCR6^+^ NK cells accurately predict how well an FNA represents the intrahepatic compartment using analytical methods. (A) Visualisation of dimension reduction of analysed lymphocyte subsets (global CD4^+^ T cells, CD8^+^ T cells, NK cells, B cells, CXCR6^±^NK cells, and CD69^+^CD103^+^/CD69^+^ CD103^−^ CD8 T_RM_ cells), using PCA for blood samples (blue) and liver biopsy samples (red) including PC1 weights. (B) Logistic regression identifies the classification boundary that best separates liver from blood (dashed line). FNA samples (green) are projected on to the PCA visualisation to determine which side of the boundary they sit. (C) Model accuracy, with 95% CIs shown, obtained by bootstrapping using lymphocytic subsets as predictors of how ‘liver-like’ an FNA sample is; the equation used to score accuracy using %CXCR6^+^ NK cells is shown. (D) Table depicting demonstration of the utility of the FNA tool using three additional FNA samples. The proportion of CXCR6^+^ NK cells, and the resulting score indicates how well these FNAs had sampled the intrahepatic immune landscape. FNA, fine needle aspirate; PCA, principal component analysis; CI, confidence interval.

First, we visualised the differences between blood and liver biopsy immune profiles using PCA, an exploratory technique projecting high dimensional data into a new smaller set of dimensions. As expected, this clearly showed that the subset composition of blood (blue) and liver (red) samples could be distinguished using the two-dimensional projection that PCA provides. Investigation of the PC1 weights implied that the frequency of the population of liver-resident NK cells drives the stratification of samples; PC2 weights are not shown as samples could be fully segregated by PC1 alone (figure 5A). We then applied logistic regression to build a model allowing separation of liver and blood samples (dashed line, figure 5A). The FNA data from all available immune parameters could then be superimposed onto the PCA to visualise their position according to the modelled cut-off (green dots, figure 5B).

To simplify this for routine reproducible longitudinal monitoring, we next used bootstrapping to test whether it was possible to select a single immune cell parameter on which to separate liver from blood. Whereas the proportion of total NK cells was a poor predictor (59% accuracy), the CD4/CD8 ratio, which is routinely available in many diagnostic laboratories for HIV monitoring, gave an improved accuracy of 89% (figure 5C). However, the use of T cell or NK cell residency markers (CXCR6^+^ NK cells or CD69^+^CD103^+ ^CD8 T_RM_ cells) allowed ~100% accuracy in the prediction of how well the intrahepatic compartment had been sampled (figure 5C). Using the equation shown or via an ‘FNA tool’ (figure 5C; see Materials and methods) investigators can obtain a colour-coded percentage score for how ‘liver-like' an individual FNA sample is. We checked the utility of this tool using a test data set of three additional FNA samples not included in the training data set. The proportion of CXCR6^+^ NK cells, and the resulting score calculated using the FNA tool, are shown in figure 5D.

## Discussion

In this study, we show that FNAs are able to sample all immune cell types tested in the liver, providing a broad and accurate profile of intrahepatic immunity. By comparing PBMCs with lymphocytes isolated from FNAs and biopsies from the same donors, we evaluate how representative FNAs are of the ‘true’ liver compartment sampled by biopsies and develop an algorithm for assessing this in future studies. Our data reveal that recently defined liver-resident subsets of T cells and NK cells, which cannot be sampled in blood, can be reproducibly identified in both FNAs and biopsies. Importantly, HBV-specific T cell responses can be found as reliably in FNAs as in biopsies, using either HLA-A2+/peptide multimers or ICS. In FNAs, viable hepatocytes can be gated and analysed in the same panel as lymphocyte markers, allowing simultaneous analysis of immune/target cell interactions. Sufficient cells can be obtained from two FNA passes for three flow cytometry panels with 16 parameters each, allowing parallel in-depth profiling of immune phenotypes, virus-specific responses and hepatocyte markers.

While liver biopsies clearly remain the gold standard for intrahepatic analysis^5^ we did not identify any immune cell subset that could not be adequately sampled by FNA. We did find that FNAs were less ‘liver-like’ in their immune composition than biopsies but, importantly, they exhibited a proportional representation of all subsets examined. Thus, a particular immune response expanded in a biopsy of one individual compared with others, was similarly relatively enriched in their FNA. This was reflected in the strong and significant correlations seen between FNA and biopsy profiles for most parameters examined. Interestingly circulating frequencies of MAITs and B cells correlated robustly with those in both FNAs and biopsies, suggesting that blood monitoring is reasonably representative of the liver for crude numbers of these cell types. However, as expected, the blood was not at all representative of intrahepatic frequencies of tissue-resident NK cells and T cells, or HBV-specific CD4 and CD8 T cells.

We used mathematical modelling to evaluate the utility of different parameters to reflect the degree to which an individual FNA was representative of the whole immune composition seen in a liver biopsy. We found that the routinely measured CD4/8 T cell ratio could be used alone to evaluate this with approximately 90% accuracy. However, quantification of the percentage of CXCR6^+^ NK cells brought accuracy up to 100% and could be relatively easily incorporated into studies to allow longitudinal standardisation of FNA samples. A further advantage of the use of CXCR6^+^ NK cells for standardisation is that they should not be affected by T cell-targeted immunotherapies (unlike the CD4/8 T cell ratio or CD69^+^CD103^+ ^CD8 T cells), one of the likely applications of FNA for longitudinal monitoring.

A novel aspect of this study was the demonstration that FNAs were capable of sampling tissue-resident subsets of T cells and NK cells from the liver. Both of these have been implicated as front-line defenders against HBV and are important therapeutic targets that cannot be monitored in the blood.^7 10 32^ The CXCR6^+ ^T-bet^lo^Eomes^hi^ subset of liver-resident NK cells was not present at significantly different proportions in FNAs and biopsies. This implies that FNAs will constitute a reliable means for following the impact of future immunotherapies on both liver-resident and liver-infiltrating components of the NK cell response. Efficient FNA sampling of intrahepatic NK cells, even the tissue-resident fraction, is in line with their putative intrasinusoidal rather than parenchymal localisation. By contrast, CD103^+^CD69^+^ CD8 T_RM_ were more abundant in biopsies than FNAs, suggesting a fraction of them may have transmigrated into the parenchyma from the vasculature, or be tethered more firmly to membrane-bound chemokine ligands, and therefore less amenable to aspiration. This concept was supported by the finding that the expression of the chemokine receptors CXCR6, and more strikingly CXCR3, was enriched on CD8 T_RM_ isolated from biopsies compared with FNAs. CXCR3 has been shown to promote the adhesion of effector cells to hepatic endothelium and their subsequent transmigration.^33^ Although our previous limited immunostaining^7^ and murine studies^34^ identified hepatic CD8 T_RM_ within the vasculature, future multiparameter immunostaining could elucidate whether some migrate into the parenchyma.

Despite the differences in frequencies of hepatic CD8 T_RM_ isolated by these two sampling methods, their expression of PD-1 and CD39 was strongly correlated. Thus prototypic CD8 T_RM_ with key features thought to underpin their survival in the liver niche, were reliably sampled by FNAs. Critically, intrahepatic HBV-specific T cells, mostly expressing residency markers CD69 ±CD103, were well sampled by FNA and biopsy. This reveals the utility of FNA for immune monitoring of patients undergoing future treatment trials for HBV cure, since the analysis of HBV-specific T cells and whether they can be augmented at the site of infection will be central to this. Prediction of a favourable response to treatment interruption using HBV-specific CD8 T cell frequencies^35^ may similarly be enhanced by the capacity of FNA to monitor the magnitude, specificity and phenotype of responses compartmentalised within the liver. Analysis of HBV-specific CD4 and CD8 T cell cytokine production will provide useful insights into boosting of local non-cytolytic antiviral capacity, whereas analysis of the therapeutic induction of a residency phenotype could inform their likely persistence to maintain functional cure.

Flow cytometric analysis of hepatocytes isolated from biopsies has been limited by their poor viability following mechanical digestion. This limitation is overcome by the use of FNAs, which do not require any processing and, we show they can allow parallel analysis of hepatocytes and lymphocytes or myeloid cells. A remaining concern is the problem of high autofluorescense of hepatocytes, requiring careful controls to optimise monoclonal antibody (mAb) staining in future studies. It has previously been shown that HBV antigens can be stained in hepatocytes from FNA;^20^ our findings pave the way for combining this with the analysis of antiviral T cell responses from the same sample, allowing ex vivo analysis of virus/host interactions compartmentalised within the liver. Our results also exemplify the possibility of FNA monitoring of PD-L1 on hepatocytes and/or liver-infiltrating myeloid cells in conjunction with PD-1 on T cells, for example, in the setting of checkpoint blockade trials.

In conclusion, we demonstrate that liver FNAs provide a useful alternative to biopsies for monitoring HBV, since they are able to concurrently provide a representative profile of key intrahepatic immune populations and hepatocytes. Next-generation flow cytometers allow around 30 parameters to be stained per well and multidimensional analysis techniques will aid visualisation of immunotherapy-induced changes in diverse immune cell subsets. Such an approach to FNA analysis has already been pioneered by Hengst *el al*, who also showed that immune profiling of FNAs is well preserved following their cryopreservation.^36^ The potential to analyse liver-resident HBV-specific T cells will be instrumental to the analysis of novel therapeutic combinations and it may even prove possible to combine this with cccDNA quantification from FNAs. The capacity to use FNAs for longitudinal monitoring of responses to therapy has already been demonstrated for HCV^18 37^ and will be an important addition to the assessment of early phase trials in HBV. Finally, our demonstration of the potential to study other aspects of immunity, underscores the broad applicability of FNAs in the monitoring of other liver diseases and infections.
