# Supplementary material for: Fine needle aspirates comprehensively sample intrahepatic immunity
Source: Gut. 2018 Nov 28;68(8):1493–503. doi: 10.1136/gutjnl-2018-317071 (PMC6691856; doi:10.1136/gutjnl-2018-317071)
Supplement: Supplementary file 1 [file gutjnl-2018-317071supp001.pdf]

ONLINE SUPPLEMENTARY MATERIAL

SUPPLEMENTARY METHODS

Patient sampling

In patients with CHB baseline hepatitis B serology was measured, including HBV DNA levels and HBV genotype where available, quantified by real-time PCR (Roche COBAS AmpliPrep/COBAS Taqman HBV test v2.0-dynamic range 20 to 1.7×10^8 IU/ml-Roche molecular diagnostics, Pleasanton, CA) and HBsAg titre (Abbott Architect). Serum was also tested for eAg and anti-HBe with a chemiluminescent microparticle immunoassay (Abbott Architect, Abbott Diagnostics, Abbot Park, IL). In patients without evidence of viral liver disease (excluded by a full non-invasive biochemical/viral liver screen), serum transaminases were measured. Histological staging of liver tissue (Ishak fibrosis stage and necroinflammatory score) was recorded in patients with CHB and the histological changes documented, confirming aetiology of liver disease, in patients without viral infection, following assessment by histopathology (Supplementary Table S1A and S1B). Prior to undertaking FNA and liver biopsy an ultrasound scan and alpha-feto protein measurement was carried out to exclude intrahepatic lesions and abnormal anatomy. Platelet count of >80,000 U/L, prothrombin time <14.5 seconds and the absence of anti-platelet therapy use for at least 7 days was confirmed prior to performing the procedures.

Phenotypic and functional analysis by flow cytometry

Cells were stained with a fixable Live/Dead dye (ThermoFisher Scientific) concurrently with saturating concentrations of surface mAbs (Table S2) in a buffer containing 50%-Brilliant Violet Buffer (BD Bioscience) and 50%-PBS for 30min at 4°C. Once stained PBMC and IHL were fixed and permeabilised, where necessary, with either Cytofix/Cytoperm (BD Bioscience), or with the FoxP3 Buffer Kit (BD Bioscience) according to manufacturer's instructions. Intracellular proteins were detected with saturating concentrations of mAbs for 30mins at 4°C in either a 0.1% saponin (Sigma-Aldrich) buffer containing 10% FBS (Sigma-Aldrich) or 1x PBS. All samples were acquired in a 0.1% saponin buffer on an X20Fortessa-SORP (BD Biosciences) and analysed in FlowJo (TreeStar).

Dextramer staining

In HLA-A2$^+$ patients with CHB infection, HBV-specific T cells were detected with HLA-A2-restricted dextramers (Immudex) of the following specificities: core 18-27 (FLPSDFFPFV), envelope 183-191 (FLLTRILTI), envelope 335-342 (WLSLLVPFV), envelope 348-357 (GLSPTVWLSV), polymerase 455-463 (GLSRYVARL) and polymerase 502-510 (KLHLYSHPI). PBMC or IHL were stained with the pool of dextramers at 37°C in 1x PBS, washed twice and left to rest for 1hr in cRPMI before further mAb staining using the methodology above. During analysis, stringent gating criteria were applied with doublet, dead and CD19$^+$ cell exclusion to minimise non-specific binding contamination. A dextramer loaded with an irrelevant peptide was used in parallel to further control for non-specific binding.

Peptide stimulation

In HLA-A2$^-$ patients with CHB infection, HBV-specific T cells were detected by ICS after an overnight stimulation with 10μg/ml or 1μg/ml overlapping peptides (pool of 15-mer peptides overlapping by 10 residues) spanning the core, envelope and polymerase regions of HBV genotype D (JPT Technologies, and Massachusetts General

Hospital Peptide Synthesis Facility) or pan-genotpyic peptide (generously provided from Gilead Life Sciences). In addition for 1 HLA-A2$^+$ patient, HBV-specifc T cell functionality was detected by ICS after overnight stimulation with 1μg/ml overlapping peptides for the 7 immunodominant peptides specific to the HLA-A2$^-$ restricted dextramers. PBMC or IHL were stimulated in the presence of 1μg/ml brefeldin-A (Sigma-Aldrich) in cRPMI for 16hrs at 37°C. HBV-specific T cells were detected by ICS for IFN-γ using the methodology as above.

SUPPLEMENTARY FIGURE LEGENDS

Figure S1: Detection of HBV-specific T cell populations by separate peptide pools in FNA and liver biopsy samples

(A) Representative flow cytometry plots showing the frequency of HBV-specific $CD3^+CD4^-CD8^+$ T cells measured by the production of IFN-γ by ICS following 16 hr stimulation with 1μg/ml of overlapping peptides spanning the core, envelope and polymerase proteins in a highly viraemic patient in matched blood, FNA and liver biopsy tissue (%IFN-γ presented minus paired unstimulated control). Representative plots showing (B) an FNA and (C) biopsy in the same patient, with ex vivo staining with a panel of HLA-A2/HBV peptide dextramers (gated using a HLA-A2 restricted dextramer control) and the frequency of HBV-specific $CD3^+CD4^-CD8^+$ T cells measured by the production of IFN-γ by ICS following overnight stimulation with OLP for the 7 immunodominant peptides specific to the HLA-A2-restricted dextramers.

Figure S2: Correlation of lymphocyte populations in blood and the intrahepatic compartment

Correlation of CD8 T (n=37), MAIT (n=20), NK (n-37) and B cells (n=36) in (A) blood compared to FNA and (B) blood compared with liver biopsy samples. (C) Correlation of CD8 T_{RM} (n=37)in blood vs. FNA and blood vs. biopsy. (D) Correlation of HBV-specific CD8 and CD4 T cells in blood vs. FNA and blood vs. biopsy (n=4). (E) Correlation of CXCR6$^+$ (n=32) and CXCR6$^+$ T-bet^{lo}Eomes^{hi} (n=10) NK cells in blood vs. FNA and blood vs. liver biopsy. p-values were determined by Spearman's rank correlation coefficient; significant changes marked with asterisks, *p<0.05; **p<0.01; ***p<0.001, ****p<0.0001.

Table S1A: HBV patients sampled

Pt. No	Sex	Age	Genotype	HBeAg	HBeAb	ALT	HBV DNA	HBsAg	Ishak Fibrosis stage	HAI Score	Disease Phase	Panel 1	Panel 2	Panel 3	Panel 4	Panel 5
Pt.1	F	28		-	+	35	4.14	4.11	1	1	eAg- CH					
Pt.2	M	31		-	+	48	3.51	4.26	0	1	eAg- CH					
Pt.3	F	44	D	-	+	15	3.84	3.80	1	4	eAg- CH					
Pt.4	M	31	A	-	+	64	3.82	3.95	0	5	eAg- CH					
Pt.5	M	50		+	-	21	1.30	2.65	0	1	eAg+ CH			Dex		
Pt.6	M	25	C	-	+	79	4.80	3.69	3	3	eAg- CH					
Pt.7	M	38		-	+	46	5.16	2.82	1	2	eAg- CH			OLP$^{(C,E)}$		
Pt.8	M	26	D	+	-	285	9.33	5.05	2	5	eAg+ CH			OLP$^{(C,E)}$		
Pt.9	M	38	D	-	+	18	3.57	3.24	0	2	eAg- CH			OLP$^{(C,E)}$		
Pt.10	M	53	B	-	+	188	5.54	2.32	1	3	eAg- CH			Dex		
Pt.11	F	31		-	+	18	3.80	3.65	1	2	eAg- CH			OLP$^{(C,E)}$		
Pt.12	F	32	D	-	+	56	1.30	4.00	0	0	eAg- CH			OLP$^{(C,E)}$		
Pt.13	M	67		-	+	27	2.40	2.65	1	3	eAg- CI			Dex		
Pt.14	M	35		-	+	39	3.19	4.42	1	2	eAg- CI					
Pt.15	M	44	C	-	+	53	1.30	2.92	0	1	eAg- CH			OLP$^{(C,E)}$		
Pt.16	M	42		-	+	13	3.73	3.78	4	6	eAg- CH			Dex		
Pt.17	M	46		-	+	27	4.16	4.39			eAg- CH			Dex		
Pt.18	M	49		-	+	33	4.89	3.35	1	4	eAg- CH					
Pt.19	F	27		-	+	27	3.56	2.58	1	2	eAg- CH			OLP$^{(C,E)}$		
Pt.20	M	32		-	+	19	4.53	4.45			eAg- CH			OLP$^{(C,E,P)}$		
Pt.21	M	24	D	+	-	279	9.14	4.86	0	5	eAg+ CH			OLP$^{(C,E,P)}$		
Pt.22	M	29	E	-	+	275	6.47	3.87	0	4	eAg- CH			OLP$^{(C,E,P)}$		
Pt.23	M	27	D	-	+	38	4.33	3.21	0	2	eAg- CH			OLP$^{(C,E,P)}$		
Pt.24	F	36	D	-	+	23	3.55	4.12	1	3	eAg- CH			OLP$^{(C,E,P)}$		
Pt.25	M	33	E	-	+	29	5.39	3.79	1	3	eAg- CH			OLP$^{(C,E)}$		
Pt.26	M	36		-	+	22	2.39	2.32	0	0	eAg- CI			Dex		
Pt.27	M	40	C	-	-	50	1.30	3.28	0	2	eAg- CH			OLP$^{(C,E,P)}$		
Pt.28	M	38		-	+	61	1.30	3.31	1	2	eAg- CH			OLP$^{(C,E)}$		

* Indicates patient sampled during nucleos(t)ide analogue therapy
^ Only blood and FNA sampled; tissue not available for IHL analysis

eAg+ CI – disease phase: eAg positive chronic infection (formerly immune tolerant)
eAg+ CH – disease phase: eAg positive chronic hepatitis (formerly immune clearance/active)
eAg- CI – disease phase: eAg negative chronic infection (formerly immune control)
eAg- CH – disease phase: eAg positive chronic hepatitis (formerly immune reactivation)

Grey shading indicates analysis undertaken; Panel 1: Lymphocyte profiling; Panel 2: Transcriptional profiling, Panel 3: HBV-specific T cell analysis (Dex: Dextramer analysis; OLP – overlapping peptide stimulation); Panel 4: Hepatocyte analysis; Panel 5: Myeloid cell profiling
Dex – Dextramer staining; OLP – stimulation with overlapping peptides (C,E,P – refer to core, envelope and polymerase peptides respectively)

Table S1B: Non-HBV patients sampled

Pt. No	Sex	Age	ALT	Histology	Panel 1	Panel 2	Panel 4	Panel 5
Pt.29	M	51	276	HH – iron overload & fibrosis				
Pt.30	M	37	68	DILI – mild inflammation				
Pt.31	F	54	85	AIH – mild inflammation				
Pt.32	F	28	168	PBC – early fibrosis				
Pt.33	F	62	48	DILI – inflammation & early fibrosis				
Pt.34	M	36	28	AIH – mild fibrosis				
Pt.35	M	17	43	PSC – mild inflammation & fibrosis				
Pt.36	M	66	50	NASH – moderate fibrosis				
Pt.37	F	56	585	DILI – moderate inflammation				
Pt.38	M	26	131	NASH – moderate inflammation				
Pt.39	M	61	148	NASH – moderate inflammation				
Pt.40	M	67	64	NASH – moderate inflammation & fibrosis				
Pt.41	M	33	213	ASH - Moderate inflammation & fibrosis				
Pt.42	M	24	15	Sickle cell disease – advanced fibrosis				
Pt.43	F	67	75	AIH – cirrhosis				

^ Only blood and FNA sampled; tissue not available for IHL analysis

AIH - Autoimmune hepatitis; ASH – Alcoholic steaotohepatitis; DILI - drug induced liver injury; HH - hereditary haemochromatosis; NASH - non-alcoholic steatohepatitis; PBC - primary biliary cholangitis; PSC - primary sclerosing cholangitis;

Grey shading indicates analysis undertaken; Panel 1: Lymphocyte profiling, Panel 2: Transcriptional profiling, Panel 4: Hepatocyte analysis, Panel 5:

TABLE S2

Antigen	Fluorochrome	Supplier	Clone
CD45	BUV805	BD Bioscience	HI30
CD45	BV711	Biolegend	HI30
CD3	BV711	Biolegend	OKT3
CD3	BUV395	BD Bioscience	UCHT1
CD19	BV786	Biolegend	SJ25C1
CD56	PE-Cy7	BD Bioscience	NCAM16.2
CD4	APC-Cy7	BD Bioscience	RPA-T4
CD4	BUV395	BD Bioscience	SK3
CD8	AlexaFluor 700	eBioscience	OKT8
CD161	APC	Miltenyi	191B8
CD161	PE	Miltenyi	191B8
Va7.2	FITC	Biolegend	3C10
CD69	PE/Dazzle594	Biolegend	FN50
CD69	BV605	Biolegend	FN50
CD103	BV605	Biolegend	Ber-ACT8
CD103	BV711	Biolegend	Ber-ACT8
CXCR6 (CD186)	BV421	Biolegend	KO41E5
CXCR6 (CD186)	PE	Biolegend	KO41E5
CXCR3 (CD183)	PerCP-Cy5.5	BD Bioscience	IC6
PD-1 (CD279)	PE	Biolegend	EH12.2H7
CD39	BV421	Biolegend	A1
T-bet	APC	eBioscience	eBio4B10
Eomes	PE-eFluor610	eBioscience	WD1928
IFNg	V450	BD Bioscience	B27
Albumin	APC	R&D Systems	188835
PD-L1 (CD274)	PE/dazzle-594	Biolegend	29E.2A3
SR-B1	PE	Biolegend	m1B9
Cytokeratin	FITC	ThermoFisher Sci.	C-04
CD14	APC-Cy7	Biolegend	M5E2
CD16	BV711	Biolegend	3G8

Lineage: CD3	FITC	eBioscience	OKT3
Lineage: CD19	FITC	Biolegend	HIB19
Lineage: CD56	FITC	Biolegend	(NCAM) MEM-118
Lineage: CD66b	FITC	Biolegend	G10F5
Lineage: CD20	FITC	Biolegend	2H7
HLA-DR	BV510	Biolegend	L243
CD123	BUV395	BD Bioscience	7G3
CD11c	BV421	Biolegend	3.9
CD68	PE	Biolegend	Y1/82A

Full details of all mAb used in the study
