# Supplementary figures and images for: Fine needle aspirates comprehensively sample intrahepatic immunity
Source: Gut. 2018 Nov 28;68(8):1493–503. doi: 10.1136/gutjnl-2018-317071 (PMC6691856; doi:10.1136/gutjnl-2018-317071)

Figure S1

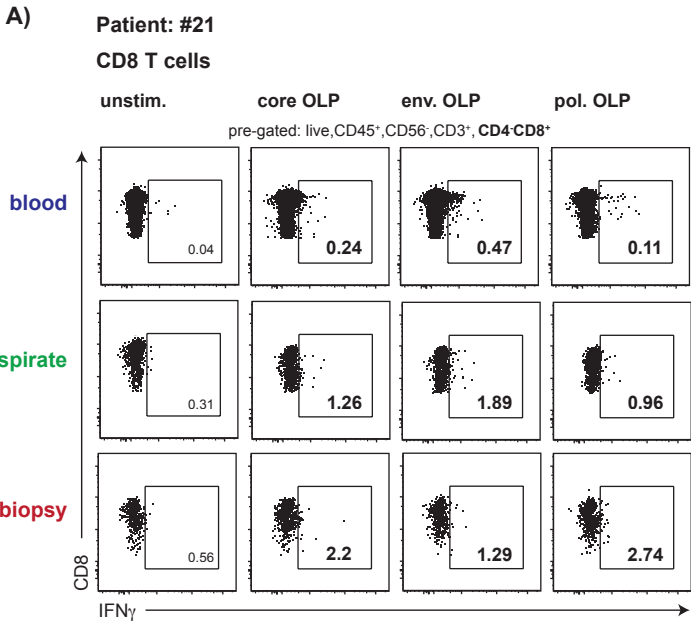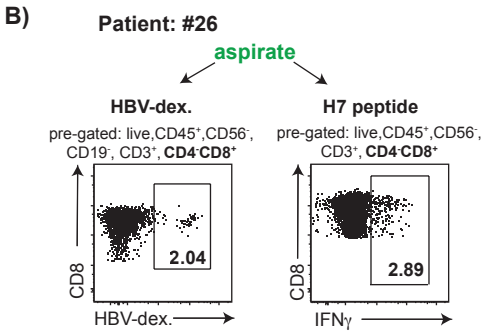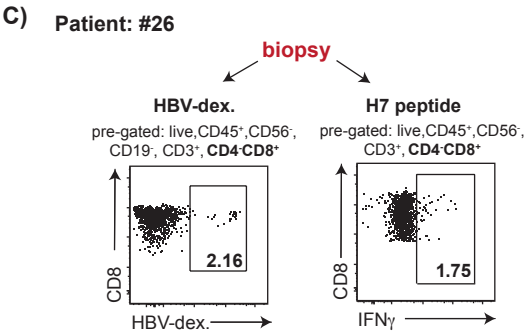

Supplement: Supplementary file 2 [file gutjnl-2018-317071supp002.pdf]

**Figure S2**

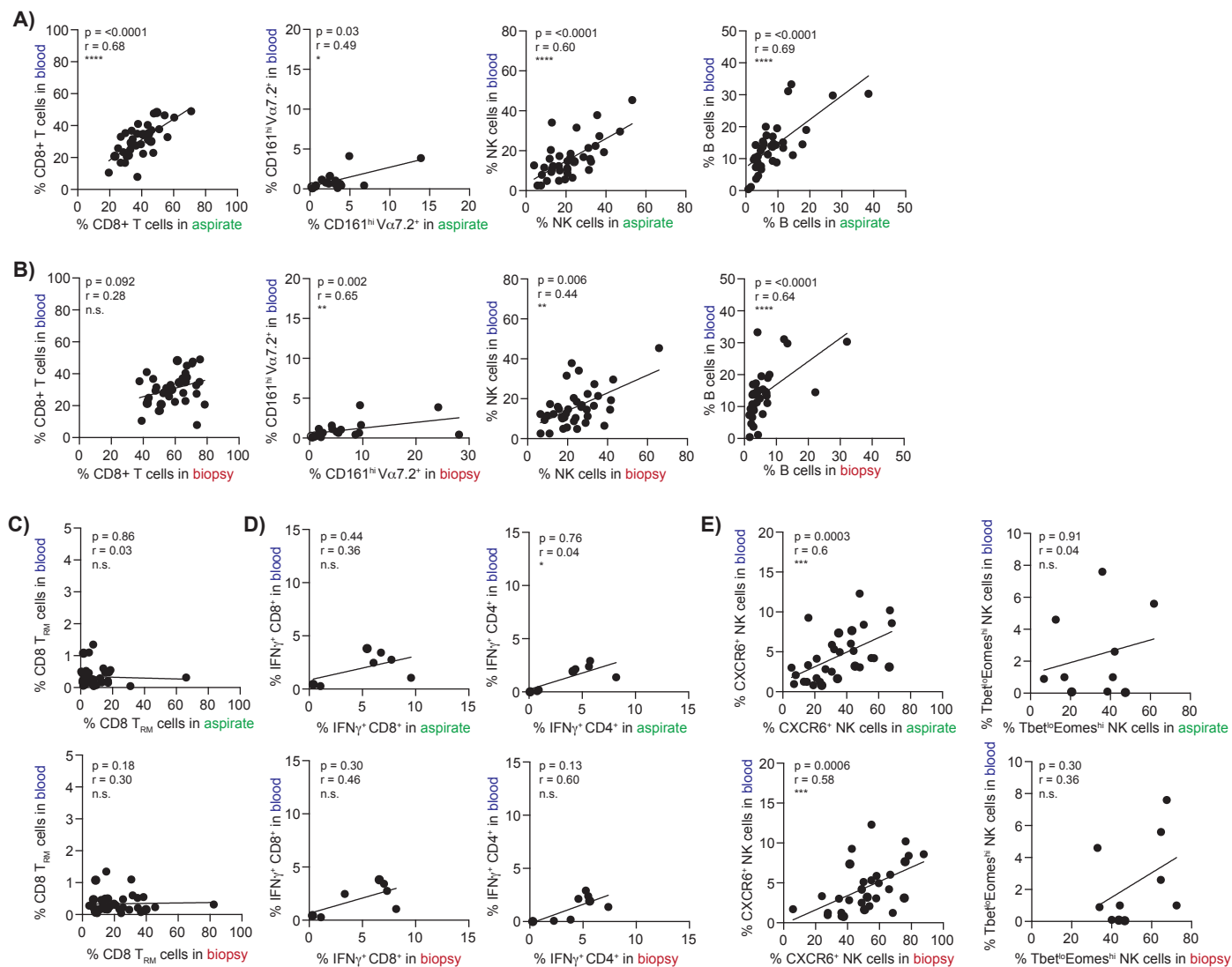

Supplement: Supplementary file 3 [file gutjnl-2018-317071supp003.pdf]
